# Vulnerable frequency as an independent prognostic factor for sudden sensorineural hearing loss

**DOI:** 10.3389/fneur.2022.962376

**Published:** 2022-09-27

**Authors:** Chaoqun Liang, Qi Fang, Hongjun Chen, Zhixian Wang, Xiangyun Qiao, Yaqi Liao, Chenxi Lv, Mo Chen, Lingxue Li, Jianming Yang

**Affiliations:** Department of Otolaryngology-Head and Neck Surgery, The Second Affiliated Hospital of Anhui Medical University, Hefei, China

**Keywords:** sudden sensorineural hearing loss (SSNHL), cochlea, predictive, prognostic, frequency

## Abstract

**Objectives:**

Sudden sensorineural hearing loss (SSNHL) is a common otology emergency in the practice. Its severe hearing impairment and prognosis impair the quality of life. Given that cochlear hair cell vulnerability is not consistent across frequencies, this study aims to investigate the impact of frequency-specific hearing loss on prognosis in SSNHL.

**Methods:**

The study included 255 patients with full-frequency SSNHL. The baseline, clinical, and hearing characteristics, as well as possible cardiovascular predictors in blood, were collected for analysis.

**Results:**

The 4,000 and 8,000 Hz hearing levels in the responder group were significantly lower than those in the non-responder group (*p* = 0.008, *p* < 0.001), while the average hearing was not (*p* = 0.081). Logistic regression showed that only vertigo (OR, 95% CI, 0.265, 0.102–0.684, *p* = 0.006) and 8,000 Hz hearing level (OR, 95% CI, 0.943, 0.916–0.971, *p* < 0.001) were strongly associated with treatment outcome.

**Conclusions:**

Compared with other frequencies, 8,000 Hz hearing level was closely related to prognosis in SSNHL. In an adjusted model, our study did not find an effect of mean hearing on prognosis in SSNHL. However, further multicenter prospective studies are needed for validation.

## Introduction

Sudden sensorineural hearing loss (SSNHL) is characterized by rapid hearing loss of at least 30 dB on at least three consecutive frequencies on an audiogram within 3 days (1). SSNHL affects 5–27 per 100,000 people annually, with 6,600 new cases reported annually in the United States (2, 3). The pathological mechanism of SSNHL is unknown, and previous studies have shown that microcirculatory disturbance, prethrombotic susceptibility, and chronic inflammation are associated with its occurrence and prognosis (4–6). The etiology of SSNHL usually varies according to its audiogram, and it is currently believed that full-frequency hearing loss has a poor prognosis and is related to the mechanism of hemodynamic disturbance (7, 8). The cochlea is a single terminal artery supplying blood, and blood circulation disorders can easily affect the function of the inner ear (9, 10). The hair cells at the base of the cochlea sense the highest sound frequencies (11), Studies have shown that the base of the cochlea is highly vulnerable and is more susceptible to ischemia and hypoxia, oxidative stress, trauma, and poisoning (12–14). Although some studies suggest that the average degree of hearing loss may affect the prognosis of SSNHL (15, 16), it is unclear whether hearing thresholds at different frequencies have a significant impact on the prognosis of SSNHL.

Given the anatomical specificity of the cochlea and the possible pathogenesis of SSNHL, in this study, we observed the clinical data of SSNHL patients to explore whether hearing thresholds at different frequencies were associated with the prognosis of SSNHL.

## Materials and methods

### Patients

We reviewed the clinical data of full-frequency SSNHL patients admitted to the head and neck surgery department of a university hospital between January 2018 and May 2022. Patients included in the study had their first visit within 14 days of the onset of SSNHL. All patients underwent a detailed physical examination, audiometry, computed tomography, and magnetic resonance imaging of the inner ear. Patients with conductive hearing loss, chronic otitis media, Meniere's disease, or retrocochlear lesions that might interfere with the diagnosis of SSNHL were excluded. At the same time, patients with recent fever and previous comorbidities such as malignant disease, mental illness, heart failure, stroke, and diabetes were excluded. To exclude the influence of the hearing level of the unaffected ear, patients with hearing loss in the unaffected ear were not included in the inclusion criteria of this study.

### Data collection

Baseline characteristics [age, sex, height, weight, body mass index (BM)], clinical characteristics (audiogram, affected side, tinnitus, ear fullness, dizziness, treatment delay), and pure-tone average (PTA) of impaired frequencies (7, 8, 17–19). The maximum value of air conductance at each frequency measured by our equipment is 100 dB. Considering the predictive role of some blood parameter markers in cardiovascular and SSNHL, we collected the pretreatment blood test results of the patients.

### Treatment and evaluation

According to the Chinese SSNHL guidelines (8), all included patients received systemic steroids (dexamethasone 10 mg/day for 5 days), ginkgo biloba, and batroxobin (10 U batroxobin for the first time and then reduced to 5 U batroxobin, once every other day, discontinue use when fibrinogen is <1 g/L). Patients were divided into two groups according to their hearing recovery after 2 weeks of treatment: responder (PTA improvement ≥30 dB, or returned to normal) and non-responder (PTA improvement <30 dB at damaged frequencies) (8).

### Statistical analysis

Statistical analysis was performed using SPSS 26.0. Continuous data are expressed as mean ± standard deviation, or median (interquartile range). Categorical data are presented as numbers (percentages), and statistical analysis used the chi-square test. Data were surveyed using the Kolmogorov-Smirnov test to determine distribution patterns. Two-sample *t*-tests were used for normally distributed quantitative data comparisons; Mann-Whitney U tests were used for non-normal quantitative data. Linear correlation to assess the association between different frequency hearing thresholds and hearing recovery. Receiver operating characteristic (ROC) curve analysis was used to assess the relationship between pre-treatment hearing thresholds at different frequencies and prognosis in SSNHL. Odds ratios (OR) and 95% confidence intervals (CI) for associations between admission hearing and SSNHL outcomes were calculated using logistic regression models. A variance inflation factor was used to check for collinearity among variables before performing multivariate logistic regression. *p* < 0.01 was considered significant for all tests. The figures were generated using GraphPad Prism 8.0.

## Results

### Baseline characteristics of SSNHL patients

After excluding 41 patients with hearing loss in the unaffected ear, a total of 255 patients with full-range SSNHL were included, as shown in Table 1. The median age of patients was 44.00 (30.00–52.00) years, and 54.5% were male. The accompanying probability of tinnitus, aural fullness, and vertigo were 94.9, 32.5, and 25.5%, respectively. The PTA of the affected ear before treatment was 75.00 (56.67–91.67) dB, and 42.0% of the patients had total deafness hearing loss. The time from onset to treatment was 3.00 (1.00–5.00) days. Averages for 0.25, 0.5, 1, 2, 4 and 8 kHz hearing are 75.00 (45.00–90.00), 75.00 (55.00–95.00), 80.00 (60.00–95.00), 75.00 (55.00–95.00), 75.00 (60.00–100.00) and 80.00 (60.00–100.00) dB, respectively. The response rate after treatment was 27.8%.

**Table 1:** Demographics, laboratory and hearing characteristics of SSNHL.

**Baseline characteristics**	**Patients (*n* = 255)**
Age, years^b^	44.00 (30.00–52.00)
Male, No. (%)^c^	139 (54.5)
Height, cm^b^	169.00 (162.00–174.00)
Weight, kg^b^	65.00 (56.00–75.00)
BMI, kg/m2^a^	23.16 ± 3.42
Systolic pressure, mmHg^a^	122.62 ± 18.33
Diastolic pressure, mmHg^b^	78.00 (70.00–86.00)
**Laboratory parameters**	
NLR^b^	2.42 (1.89–3.66)
MLR^b^	0.23 (0.18–0.30)
PLR^b^	118.75 (92.86–159.15)
ALB, g/L^a^	43.45 ± 4.47
FIB, g/L^b^	2.46 (2.12–2.85)
**Hearing characteristics**	
Left, No. (%)^c^	138 (54.1)
Other aural symptom, No. (%)^c^	
Tinnitus	242 (94.9)
Vertigo	65 (25.5)
Aural fullness	83 (32.5)
Shapes of audiogram, No. (%)^c^	
Flat type	148 (58.0)
Profound loss	107 (42.0)
Responder, No. (%)^c^	71 (27.8)
Time to treatment, days^b^	3.00 (1.00–5.00)
PTA, dB^b^	75.00 (56.67–91.67)
PT (250 Hz), dB^b^	75.00 (45.00–90.00)
PT (500 Hz), dB^b^	75.00 (55.00–95.00)
PT (1000 Hz), dB^b^	80.00 (60.00–95.00)
PT (2000 Hz), dB^b^	75.00 (55.00–95.00)
PT (4000 Hz), dB^b^	75.00 (60.00–100.00)
PT (8000 Hz), dB^b^	80.00 (60.00–100.00)

a The values are given as mean ± standard deviation.

b The values are given as median with its interquartile range (25–75th) in parentheses.

c The values are given as the number of cases and the percentage in parentheses; BMI, body mass index; NLR, neutrophil to lymphocyte ratio; MLR, monocyte to lymphocyte ratio; PLR, platelet to lymphocyte ratio; ALB, albumin; FIB, fibrinogen; PTA, pure tone average; PT, pure tone.

### Comparison of different frequency characteristics of responder and non-responder groups

Hearing recovery patients were divided into Responder (*n* = 71) and Non-responder (*n* = 184), as shown in Table 2. There were no significant differences in baseline characteristics between the two groups (all *p* > 0.05). There was no significant difference in the affected side, audiogram, accompanying tinnitus, aural fullness, and treatment time after the onset between the two groups (all *p* > 0.05). The number of vertigo patients in the responder was significantly lower than that in the non-responder (*p* < 0.05). In terms of hearing at various frequencies, the hearing loss levels of the non-responder at 4,000 and 8,000 Hz were 80.00 (58.50–100.00) and 87.50 (65.00–100.00) dB, which were significantly higher than those of the responder [70.00 (60.00–85.00) and 70.00 (50.00–80.00) dB] (*p* < 0.05). In the comparison of blood parameters between the two groups, the albumin level in the responder was significantly higher than that in the non-responder, while the level of fibrinogen was the opposite (all *p* < 0.05). There was no significant difference between the two groups in some other possible cardiovascular validation markers (all *p* > 0.05).

**Table 2:** Comparison of prognosis between responder and non-responder.

	**Responder (*n* = 71)**	**Non-responder (*n* = 184)**	***P*** **-value**
**Baseline characteristics**			
Age, years^b^	38.00 (30.00–51.00)	46.00 (29.25–53.00)	0.104
Male, No. (%)^c^	42 (59.2)	97 (52.7)	0.355
Height, cm^b^	170.00 (163.00–175.00)	167.50 (162.00–174.00)	0.559
Weight, kg^b^	62.00 (56.00–75.00)	65.00 (57.25–75.00)	0.473
BMI, kg/m^2^^a^	22.60 ± 3.40	23.387 ± 3.41	0.108
Systolic pressure, mmHg^b^	120.00 (109.00–130.00)	121.50 (111.00–135.00)	0.136
Diastolic pressure, mmHg^b^	75.00 (68.00–84.00)	78.00 (71.00–86.00)	0.178
**Laboratory parameters**			
NLR^b^	2.42 (1.89–3.65)	2.43 (1.89–3.78)	0.795
MLR^b^	0.23 (0.18–0.28)	0.23 (0.18–0.30)	0.556
PLR^b^	133.33 (96.39–166.05)	112.94 (92.65–147.88)	0.066
ALB, g/L^a^	44.82 ± 3.96	42.92 ± 4.55	**0.002**
FIB, g/L^b^	2.30 (2.00–2.70)	2.50 (2.18–2.88)	**0.013**
**Hearing characteristics**			
Left, No. (%)^c^	36 (50.7)	90 (48.9)	0.798
Other aural symptom, No. (%)^c^			
Tinnitus	69 (97.2)	173 (94.0)	0.477
Vertigo	6 (8.5)	59 (32.1)	**< 0.001**
Aural fullness	28 (39.4)	55 (29.8)	0.145
Shapes of audiogram, No. (%)^c^			0.054
Flat type	48 (67.6)	100 (54.3)	
Profound loss	23 (32.4)	84 (45.7)	
Time to treatment, days^b^	3.00 (1.00–4.00)	3.00 (1.00–6.00)	0.077
PTA, dB^b^	70.00 (59.17–82.50)	77.50 (53.58–98.33)	0.065
PT (250 Hz), dB^b^	70.00 (50.00–85.00)	75.00 (45.00–95.00)	0.077
PT (500 Hz), dB^b^	70.00 (65.00–85.00)	75.00 (51.00–100.00)	0.321
PT (1000 Hz), dB^b^	75.00 (70.00–85.00)	80 (55.00–100.00)	0.788
PT (2000 Hz), dB^b^	75.00 (60.00–85.00)	75.50 (51.25–100.00)	0.381
PT (4000 Hz), dB^b^	70.00 (60.00–85.00)	80.00 (58.50–100.00)	**0.002**
PT (8000 Hz), dB^b^	70.00 (50.00–80.00)	87.50 (65.00–100.00)	**< 0.001**

a The values are given as mean ± standard deviation.

b The values are given as median with its interquartile range (25–75th) in parentheses.

c The values are given as the number of cases and the percentage in parentheses; BMI, body mass index; NLR, neutrophil to lymphocyte ratio; MLR, monocyte to lymphocyte ratio; PLR, platelet to lymphocyte ratio; ALB, albumin; FIB, fibrinogen; PTA, pure tone average; PT, pure tone. The bold values mean *P*-value < 0.05.

### Association between different frequencies and hearing recovery

**Figure 1:**
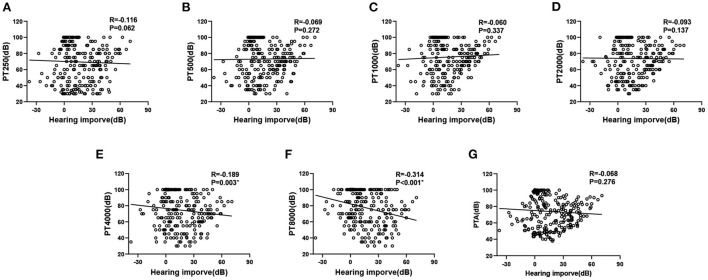
Hearing thresholds vs. hearing improvement. Hearing thresholds vs. hearing improvement. **(A–G)** represent the relationship between PT250, PT500, PT1000, PT2000, PT4000, PT8000 and PTA and hearing improve, respectively.

**Figure 2:**
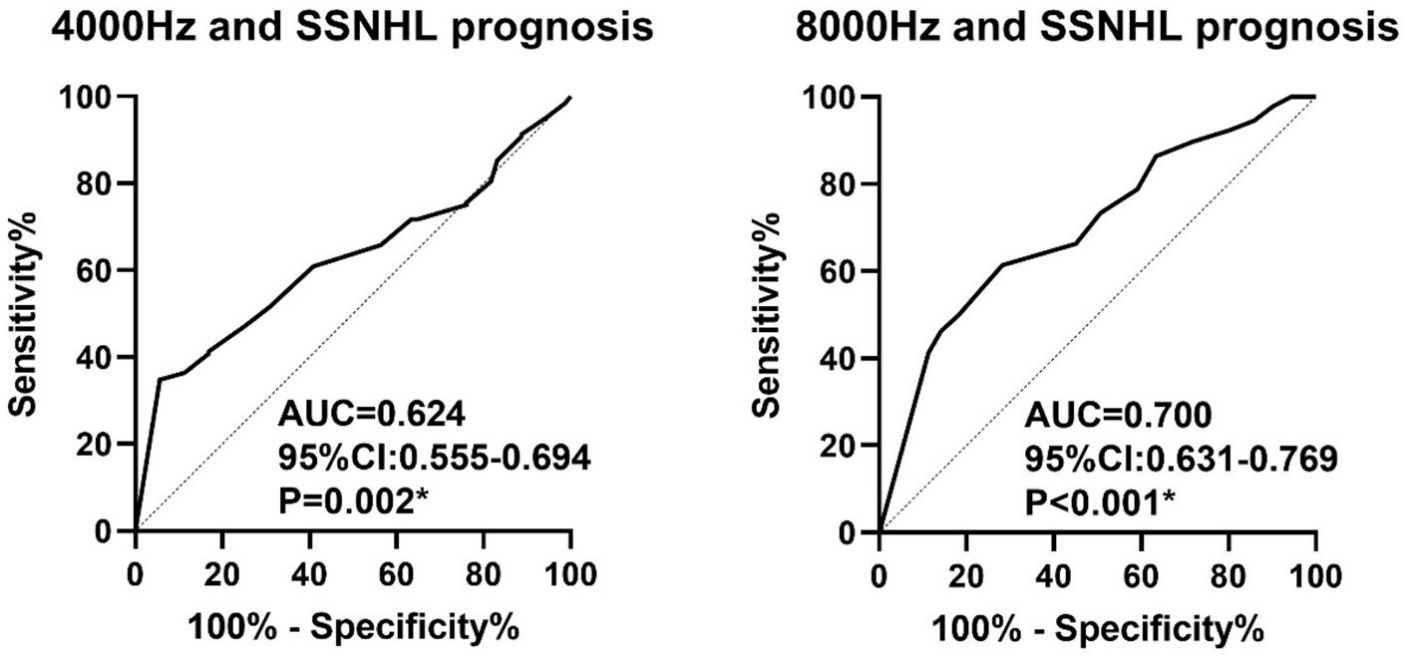
Receiver operating characteristic curve analysis of 4,000 and 8,000 Hz hearing levels for the prediction of the outcome of SSNHL.

We performed a linear correlation analysis and plotted a scatterplot of hearing thresholds at different frequencies vs. hearing improvement (Figure 1). When hearing loss was considered as a continuous variable, pre-treatment hearing thresholds at 4,000 and 8,000 Hz were negatively correlated with hearing improvement (*r* = – 0.189, *p* = 0.003, and *r* = – 0.314, *p* < 0.001, respectively), while other frequencies and PTA were not significantly correlated with hearing improvement (all *p* > 0.05). ROC curve analysis of SSNHL results showed that the areas under the curve at 4,000 and 8,000 Hz before treatment were 0.624 (95% CI, 0.555–0.694) and 0.700 (95% CI, 0.631–0.769), respectively (Figure 2). This suggests that high-frequency hearing, but not PTA, may be a predictor of poor prognosis in full-frequency SSNHL.

In univariate logistic regression analysis, the OR of SSNHL prognosis and parameters are shown in Table 3. We found that ALB, fibrinogen, vertigo, duration of treatment, and hearing at 4,000 and 8,000 Hz were associated with SSNHL treatment outcomes. Variables with test level < 0.1 in univariate analysis were included in multivariate logistic regression. A variance inflation factor was used to confirm that all parameters were non-collinear before multivariate analysis. After adjusting for all other significant predictors, vertigo and 8,000 Hz hearing remained independent predictors, with adjusted OR of 0.265 (0.102–0.684, *p* = 0.006), and 0.943 (0.916–0.971, *p* < 0.001), respectively. These results suggest that the presence of vertigo and higher hearing loss at 8,000 Hz are independent factors for poor prognosis in SSNHL.

**Table 3:** Potential factors associated with hearing recovery.

	**Univariate analysis**		**Multivariate analysis**	
	**OR (95% CI)**	***P*** **-value**	**OR (95% CI)**	***P*** **-value**
**Predictor: outcome of disease**				
Age, years	0.985 (0.967–1.004)	0.133		
Male, ref. (female)	1.299 (0.746–2.262)	0.355		
Height, cm	1.014 (0.979–1.050)	0.445		
Weight, kg	0.991 (0.969–1.014)	0.438		
BMI, kg/m^2^	0.934 (0.859–1.015)	0.109		
Systolic pressure, mmHg	0.988 (0.973–1.004)	0.132		
Diastolic pressure, mmHg	0.990 (0.967–1.013)	0.374		
NLR	0.917 (0.790–1.064)	0.253		
MLR	0.616 (0.056–6.847)	0.694		
PLR	1.003 (0.999–1.008)	0.182		
ALB, g/L	1.113 (1.039–1.193)	**0.002**	1.082 (0.998–1.172)	0.055
FIB, g/L	0.573 (0.348–0.944)	**0.029**	0.679 (0.388–1.188)	0.679
Left, ref. (right)	0.967 (0.559–1.675)	0.905		
Tinnitus	2.194 (0.474–10.154)	0.315		
Vertigo	0.196 (0.080–0.477)	**< 0.001**	0.265 (0.102–0.684)	**0.006**
Aural fullness	1.527 (0.863–2.704)	0.146		
Flat type, ref. (profound)	1.753 (0.986–3.117)	0.056	1.138 (0.341–3.792)	0.834
Time to treatment, days	0.886 (0.792–0.992)	**0.036**	0.918 (0.806–1.045)	0.194
PTA, dB	0.987 (0.973–1.002)	0.081	1.031 (0.986–1.079)	0.182
PT (250 Hz), dB	0.992 (0.981–1.004)	0.187		
PT (500 Hz), dB	0.997 (0.985–1.009)	0.627		
PT (1,000 Hz), dB	1.004 (0.991–1.018)	0.515		
PT (2,000 Hz), dB	0.998 (0.985–1.011)	0.744		
PT (4,000 Hz), dB	0.983 (0.970–0.995)	**0.008**	1.016 (0.979–1.055)	0.408
PT (8,000 Hz), dB	0.967 (0.954–0.982)	**<0.001**	0.943 (0.916–0.971)	**<0.001**

BMI, body mass index; NLR, neutrophil to lymphocyte ratio; MLR, monocyte to lymphocyte ratio; PLR, platelet to lymphocyte ratio; ALB, albumin; FIB, fibrinogen; PTA, pure tone average; PT, pure tone; OR, odds ratio; CI, confidence interval. The bold values mean *P*-value < 0.05.

## Discussion

In this study, we found that vertigo and initial hearing at 8,000 Hz were associated with hearing recovery in patients with SSNHL. To assess the role of each frequency threshold in the prognosis of SSNHL, this study included SSNHL patients with full-frequency hearing impairment. Considering the roles of inflammation and hemodynamics in patients with full-frequency SSNHL, we included some possible blood predictors for analysis. Our results suggest that it's hearing at 8,000 Hz, but not other frequencies, that predicts full-frequency SSNHL.

There are many hypotheses about the pathogenesis of SSNHL: infectious, vascular, inflammatory, immune, degenerative, etc., (20, 21). It is known that age, vertigo, and the time to delay treatment after onset are closely related to the degree of recovery of SSNHL (22–24). In recent years, more and more studies have suggested that cardiovascular factors, hemorheology, and oxidative stress are related to the occurrence and prognosis of SSNHL (25, 26), specific inflammatory and cardiovascular markers have also been shown to be related to the outcome of SSNHL (18, 27–30). However, whether this association is directly related to SSNHL is debatable. In the present study, we included possible inflammatory and cardiovascular markers, although in univariate analysis we found that ALB and FIB but not other parameters may provide guidance on the prognosis of SSNHL. However, we did not find this correlation in multivariate analysis, which seems to further explain that vascular factors further influence the occurrence and prognosis of SSNHL by affecting inner ear hair cell function. Similarly, several studies have shown that the shorter the delayed treatment time, the better the hearing prognosis (19, 31). This study did not find that treatment time had an important effect on hearing recovery, which may be based on two aspects: 1. The population included in this study followed the guidelines for the first visit within 14 days, which is relatively loose. 2. This study defines hearing recovery as hearing improvement ≥30 dB. This study suggests that patients with vertigo may have a worse prognosis, which is consistent with previous studies (23). Vestibular dysfunction significantly associated with severe hearing loss (32). Studies have reported that the vestibule as an end-organ has better collateral vascular supply than the cochlea (33, 34), so the presence of vertigo often predicts extensive inner ear damage and a poorer prognosis.

The cochlea consists of three adjacent membranous tubes, shaped like a snail, surrounded by a bony shell (35, 36). Frequency-specific hair cell receptors transduce acoustic signals in the cochlea, and these hair cell receptors map within the cochlea, creating a tonotopic structure with the lowest frequencies at the top of the cochlea and the highest frequencies at the base of the cochlea (11, 36). As an end-organ supplied by a single arterial blood supply, the cochlea lacks collateral circulation, making it susceptible to hemodynamic effects (15). Studies have shown that various vascular injuries and oxidative stress have a more significant impact on the base of the cochlea, so cochlear damage tends to spread from the base to the top (13). Based on the penetration of the round window membrane, infections outside the inner ear may cause cochlear damage even if the infection does not enter the labyrinth. Studies have shown that middle ear infections can damage the basal corner of the cochlea (37). A prognostic analysis of SSNHL based on inflammation levels also showed that cochlear ischemic changes with high levels of inflammation can make high-frequency hearing levels more vulnerable and difficult to recover from damage (38). Therefore, the base of the cochlea is very fragile.

Audiograms are thought to correlate with prognosis in SSNHL, with low-frequency decreased SSNHL having a favorable prognosis and full-frequency hearing decreased poor prognosis (8, 39). In our study, this effect was not found in an adjusted multivariate analysis. After adjusting for possible influencing factors such as age, concomitant symptoms, cardiovascular inflammatory markers, and audiograms, we found that hearing at 8,000 Hz mainly affects the prognosis of SSNHL. Several studies have shown that when vascular, endocrine and immune dysfunction accompany hearing loss, high-frequency hearing impairment first appears, of which 8,000 is common (40–45). In a per-frequency predictive model, high-frequency hearing loss in SSNHL patients was more difficult to recover than other frequencies, and this correlation was more pronounced at 8,000 Hz (46).

To our knowledge, this is the first study that adjusted for multiple factors and used a single frequency as an influencing factor to predict SSNHL and prognosis. Based on the vulnerability characteristics of specific frequencies of the cochlea and the diagnostic criteria for SSNHL, it is logical to predict the prognosis of hearing loss by the vulnerable frequencies before treatment. This study has some limitations. First, as a preliminary study, the reproducibility of the results needs to be validated in a multicenter prospective study. Then, as an adjustment for confounders, we included only blood parameters known to have predictors in SSNHL into the analysis, and future studies should consider and incorporate other possible cardiovascular predictors. Finally, we have a short follow-up period. Although several current literatures suggest that the long-term follow-up hearing changes are 4–5 dB (47–49), which is not higher than the test error of the pure-tone hearing threshold clearly stated in the AAO-HNS guidelines (1). In other words, whether this level of hearing improvement is really clinically meaningful remains to be viewed rationally.

## Conclusion

In this study, we explored the relationship of individual frequencies to SSNHL hearing improvement. Unlike previous studies, our study demonstrated that initial 8,000 Hz hearing and not other frequencies were independently associated with hearing improvement. The presence of vertigo and higher hearing at 8,000 Hz may be risk factors for poor prognosis in SSNHL, and these patients may require early combination therapy to increase the likelihood of hearing recovery.

## Data availability statement

The raw data supporting the conclusions of this article will be made available by the authors, without undue reservation.

## Ethics statement

The studies involving human participants were reviewed and approved by the Ethics Committee of the Second Hospital of Anhui Medical University: YJ-YX2019-039. The patients/participants provided their written informed consent to participate in this study.

## Author contributions

CL and QF: built the models, analyzed the data, and drafted the manuscript. HC, ZW, CL, and LL: collect and filter data. HC, XQ, YL, and MC: participated in manuscript editing. JY: reviewed the manuscript and support for this assistance was funded. All authors contributed to the article and approved the submitted version.

## Funding

The study was supported by funding from the Incubation Program of National Natural Science Foundation of China (Grant No. 2019GMFY06).

## Conflict of interest

The authors declare that the research was conducted in the absence of any commercial or financial relationships that could be construed as a potential conflict of interest.

## Publisher's note

All claims expressed in this article are solely those of the authors and do not necessarily represent those of their affiliated organizations, or those of the publisher, the editors and the reviewers. Any product that may be evaluated in this article, or claim that may be made by its manufacturer, is not guaranteed or endorsed by the publisher.
